# Diagnostic and ethical challenges in disorders of consciousness and locked-in syndrome: a survey of German neurologists

**DOI:** 10.1007/s00415-012-6459-9

**Published:** 2012-03-10

**Authors:** Katja Kuehlmeyer, Eric Racine, Nicole Palmour, Eva Hoster, Gian Domenico Borasio, Ralf J. Jox

**Affiliations:** 1 Institute of Ethics, History and Theory of Medicine, University of Munich, Lessingstrasse 2, 80336 Munich, Germany; 2 Neuroethics Research Unit, Institut de recherches cliniques de Montréal (IRCM), 110 avenue des Pins Ouest, Montreal, QC H2W 1R7 Canada; 3 Department of Medicine and Department of Social and Preventive Medicine, Université de Montréal, Montreal, Canada; 4 Departments of Neurology and Neurosurgery, Medicine and Biomedical Ethics Unit, McGill University, Montreal, Canada; 5 Institute of Medical Informatics, Biometry, and Epidemiology, University of Munich, Munich, Germany; 6 Service de Soins Palliatifs, Centre Hospitalier Universitaire Vaudois (CHUV), University of Lausanne, Lausanne, Switzerland

**Keywords:** End-of-life decisions, Limitation of life-sustaining treatment, Diagnosis, Vegetative state, Minimally conscious state

## Abstract

Diagnosis and decisions on life-sustaining treatment (LST) in disorders of consciousness, such as the vegetative state (VS) and the minimally conscious state (MCS), are challenging for neurologists. The locked-in syndrome (LiS) is sometimes confounded with these disorders by less experienced physicians. We aimed to investigate (1) the application of diagnostic knowledge, (2) attitudes concerning limitations of LST, and (3) further challenging aspects in the care of patients. A vignette-based online survey with a randomized presentation of a VS, MCS, or LiS case scenario was conducted among members of the German Society for Neurology. A sample of 503 neurologists participated (response rate 16.4%). An accurate diagnosis was given by 86% of the participants. The LiS case was diagnosed more accurately (94%) than the VS case (79%) and the MCS case (87%, *p* < 0.001). Limiting LST for the patient was considered by 92, 91, and 84% of the participants who accurately diagnosed the VS, LiS, and MCS case (*p* = 0.09). Overall, most participants agreed with limiting cardiopulmonary resuscitation; a minority considered limiting artificial nutrition and hydration. Neurologists regarded the estimation of the prognosis and determination of the patients’ wishes as most challenging. The majority of German neurologists accurately applied the diagnostic categories VS, MCS, and LiS to case vignettes. Their attitudes were mostly in favor of limiting life-sustaining treatment and slightly differed for MCS as compared to VS and LiS. Attitudes toward LST strongly differed according to circumstances (e.g., patient’s will opposed treatment) and treatment measures.

## Introduction

The vegetative state (VS) and the minimally conscious state (MCS) are conditions that result from severe traumatic or non-traumatic brain injury, referred to as disorders of consciousness (DOC). In the VS, patients are awake, but do not show any signs of awareness, as judged by responsiveness [1, 2]. Recently, the European Task Force on DOC proposed the term “unresponsive wakefulness syndrome” (UWS) as a superior alternative to VS [3]. In the MCS, patients display limited, but reproducible evidence of awareness without having the ability to communicate reliably [4]. Several studies have revealed misdiagnosis rates of 40%, in cases in which clinical bedside examination was compared to expert assessment or neurobehavioral testing [5–7]. Misdiagnosed patients were presumed to be in VS, but after a reassessment they were categorized as being in either a MCS or locked-in syndrome (LiS), where the patient is fully aware, has quadriplegia and aphonia or severe hypophonia, but is usually capable of communication by eye movements or blinking [8, 9]. Inaccurate diagnosis is both a medical and an ethical problem. It biases prognostication and therapeutic strategy, and may lead to flawed decisions to withdraw or withhold life-sustaining treatment (LST) [2, 10].

The ethical justification of administering LST in these patients is a matter of intense ethical and societal debate, specifically with regard to the use of artificial nutrition and hydration, as in the public cases of Terri Schiavo and Eluana Englaro [11, 12]. Most surveys that investigated physicians’ attitudes toward LST in DOC patients were conducted before the MCS was defined as a separate diagnostic category in 2002 [13–16]. A recently published European survey found that the diagnosis of VS or MCS influences ethical attitudes toward LST [17]. This survey, however, targeted a heterogeneous convenience sample of attendees at scientific conferences and asked for attitudes toward LST for patients in chronic VS or MCS (>1 year). To our knowledge, no survey has investigated physicians’ attitudes toward LST for patients in the LiS. A Japanese study used case vignettes to study physicians’ attitudes toward LST in VS patients, but the patient’s diagnosis was always provided. To our knowledge, no survey has used case vignettes instead of diagnostic terms to examine the participants’ attitudes toward LST. By means of a web-based survey, we wanted to examine the attitudes of German neurologists regarding ethical issues in DOC and the LiS, and to assess the application of their diagnostic knowledge to a case vignette. We aimed to answer the following research questions: (1) how accurately do neurologists apply the diagnostic categories VS, MCS or LiS to hypothetical cases? (2) Do neurologists’ attitudes toward LST for these patients differ according to the diagnosis of the patient? (3) Which ethically relevant aspects do neurologists evaluate as being the most challenging in the care of DOC and LiS patients?

## Methods

### Questionnaire

We developed a 37-item questionnaire in English. Three case vignettes (see Table 1), at the beginning of the questionnaire, were drafted by a neurologist (R.J.J) based on clinical consensus guidelines, and revised and verified by an international scientific advisory board of neurological DOC experts. The cases were presented randomly; each participant activating the link to the survey website received only one case and had an equal chance of getting one of the three cases.

**Table 1:** Case vignettes presented randomly to participants

*Case 1* ^a^ A 33-year-old man had a cardiac arrest with delayed resuscitation 4 months ago. Currently, he shows brainstem and spinal reflex movements, but no sign of purposeful movement. His eyes are open for several hours a day, but do not fixate objects or follow them when they move. He does not react consistently to verbal commands or questions. Sometimes a delayed stiffening of the legs and grimacing can be observed in reaction to sounds. He can breathe on his own
*Case 2* ^b^ A 35-year-old woman suffered a severe asthma attack with respiratory failure 4 months ago, causing severe brain injury. Currently, she shows brainstem and spinal reflexes and a severe spasticity, but no signs of purposeful movement. She does not need any breathing assistance. Her eyes are open for several hours a day, fixate objects and follow the nurses when they move around her. She does not react consistently to verbal commands or questions. When she is visited by her mother, she always seems more alert, and when her mother talks to her, she often smiles and utters single words. This does not happen when other persons talk to her
*Case 3* ^c^ A 36-year-old man had a brain stem hemorrhage 4 months ago. In the meantime he could be weaned from the ventilator. He does not move his limbs in any way and suffers from severe spasticity. During the day, his eyes are open for several hours. He consistently follows the command to blink once or twice, or to move his eyes up and down. A verbal utterance or groaning has not been observed

^a^Correct diagnosis: vegetative state (VS)

^b^Correct diagnosis: minimally conscious state (MCS)

^c^Correct diagnosis: locked-in state (LiS)

After the presentation of the case, participants were asked: “if you had to assess the described case without detailed behavioral testing or technical diagnostic investigations, which diagnosis do you think fits best to the case?” Participants could choose among five diagnostic categories (VS, MCS, LiS, brain death, and coma) or give an alternative diagnosis in an open text field. No definitions of the VS, MCS, or LiS were provided. Participants were asked how certain they were on a numeric rating scale (NRS) (0–10, 10 = extremely certain) about the diagnostic category that they assigned to the case. They were also asked to estimate the patient’s functional outcome (“What do you think will be the patient’s functional outcome in 6 months as measured by the modified Rankin scale?”). To assess the neurologists’ conceptual understanding underlying the diagnosis, they were asked to judge which cognitive, emotional and behavioral capabilities such a patient has, choosing from a given list. Furthermore, the participants should estimate the quality of life of such a patient on a NRS, including the options of “no quality of life” and “I don’t feel able to rate the patient’s quality of life.” Physicians’ attitudes toward limiting LST were elicited by the following request: “Please specify: In the prior case life-sustaining treatment should be limited (a) never, (b) always, or (c) under certain circumstances.” If participants chose “always” or “under certain circumstances,” they had to specify these on given lists of ten different circumstances. If the circumstances did not apply to the case (like recovery of consciousness to the LiS) participants could choose alternatively “does not apply.” In addition, participants were asked which specific treatment measures they would consider limiting. Finally, they were asked to rate the extent to which they find 13 ethically relevant aspects of caring for DOC patients challenging on a NRS. We asked for participants’ characteristics such as gender, age, work environment, professional experience, and religion. Religion was analyzed according to religious practice and spiritual beliefs.

The questionnaire was pre-tested by five experts (four neurologists and a medical ethicist) and modified accordingly. We translated the final questionnaire into German using backward-forward translation [18]. Two German native speakers, who were involved in constructing the English survey (K.K., R.J.J.), translated the questionnaire independently from one another into German and a native English speaker translated the survey back into English. Inconsistencies were identified and led to a refinement of both the original English and the translated German version. Online formatting was done with Survey Monkey software (Survey Monkey, Portland, OR, USA). The German questionnaire can be accessed by the link https://www.surveymonkey.com/s/UmfrageuberBewusstseinsstorungen (accessed 31 January 2012) without the need to type in a code.

### Data collection

The study was approved by the research ethics committee of the local medical faculty. To include a representative cohort of German neurologists, we contacted the German Society for Neurology (Deutsche Gesellschaft für Neurologie), which facilitated the distribution of the survey link. Members of the society are physicians with a license to practice medicine and a small number (~0.1%) are medical students. Out of 6,673 members, we contacted all 3,073 members from whom the society had e-mail addresses and invited only physicians to participate. In the initial contact e-mail, we explained the purpose, objectives, and content of the study (medical and ethical aspects, diagnosis, prognosis, and treatment decisions for patients with disorders of consciousness), the voluntariness and data protection rules, the time it might take to fill out the questionnaire (10–20 min), a deadline for the participation, and provided the link to the survey website. Members with invalid addresses were excluded. The study was powered to detect a 15% difference in the attitudes toward limiting LST among the three cases, with a probability of 80%. The data were gathered within a 4-week period from July to August 2011. To encourage participation we offered an opportunity to participate in a lottery, consisting of six prizes with a total value of €1,500. After 3 weeks, we sent a reminder and prolonged the participation period for an additional week. Data were gathered anonymously, and participants gave their informed consent.

### Statistical analysis

Participants who made errors diagnosing the patients in the case vignettes were excluded from the analysis of their attitudes and their evaluation of challenges, because it was unclear whether they answered the remaining questions according to the vignette description or according to their inaccurate choice of diagnosis.

Data were downloaded from Survey Monkey and imported into IBM SPSS 19 statistics software. Pearson’s *χ* ^2^ test was performed to assess differences between categories. For numerical or ordinal data, the Mann-Whitney *U* test was applied to compare two groups, and the Kruskal-Wallis *H* test was performed to compare three (or more) groups. Binary logistic regression analyses were used to examine associations between predictor variables and the accuracy of diagnosis, or the attitude toward limiting LST. Results were considered significant if *p* < 0.05, and a trend to significance was reported if *p* < 0.10. Following the recommendations of Perneger, *p* values are descriptive and were not adjusted for multiple comparisons [19].

## Results

### Cohort and sample

Of the 3,073 members that were contacted, 517 participated in the online survey. Some society members mentioned their reasons for not participating: They either did not provide care for patients at all (due to retirement, to other professions, or being heads of the units or institutes), or they did not take care of patients with DOC. The 517 participants were randomly assigned to the VS (*n* = 175), MCS (*n* = 176), and LiS (*n* = 166) case. Fourteen participants did not complete the questionnaire: 7 were assigned to the VS case, 5 to the MCS case, and 2 to the LiS case. A sample of 503 neurologists completed the online survey (response rate: 16.4%); 168 participants (33%) filled out the questionnaire with the VS case, 171 (34%) the MCS-based version, and 164 (33%) the LiS-based questionnaire. The cohort of 3,073 members with valid e-mail addresses was representative for all members (6,673) according to age (members: mean 44, standard deviation (SD) 10, range 25–94, and cohort: mean 45, SD 9, range 25–87) and region of practice, but not gender. A lower percentage of women (28%) was invited to participate than the actual percentage of women in the society (38%). We analyzed whether gender had a significant influence on physicians’ diagnostic accuracy or their attitudes toward limiting LST and describe these results in the respective sections. The demographic and professional characteristics of the participants are presented in Table 2. The sample was representative for the society according to age (mean 44, SD 9, range 27–81).

**Table 2:** Demographic and professional characteristics of participants (*n* = 503)

Age (years), median; 1st 3rd quartile (range)	43; 38, 49 (27–81)
Experience (years)	17; 11, 21 (<1–49)
Gender, *n* (%) (*n* = 31 missing)	
Female	140 (30)
Male	332 (70)
Primary discipline, *n* (%) (*n* = 16 missing)	
Neurology	479 (98)
Others (e.g., anesthesiology, psychiatry)	8 (2)
Health care setting, *n* (%)^a^	
In-patient care	370 (74)
Out-patient care	173 (34)
Kind of care, *n* (%)^a^	
Acute care	216 (43)
Rehabilitation care	107 (21)
Long-term care	39 (8)
Professional experience with VS patients, *n* (%) (*n* = 25 missing)	
0 cases	15 (3)
≤20 cases	261 (55)
>20 cases	202 (42)
Professional experience with MCS patients, *n* (%) (*n* = 40 missing)	
0 cases	39 (8)
≤20 cases	249 (54)
>20 cases	175 (38)
Professional experience with LiS patients, *n* (%) (*n* = 32 missing)	
0 cases	54 (11)
≤20 cases	356 (76)
>20 cases	61 (13)
Religious practice, *n* (%) (*n* = 25 missing)	
Practicing religion	250 (52)
Not practicing religion	228 (48)
Spiritual beliefs, *n* (%) (*n* = 29 missing)	
Spiritual beliefs	317 (67)
No spiritual beliefs	157 (33)

^a^Multiple answers permitted

### Application of diagnostic knowledge

**Fig. 1:**
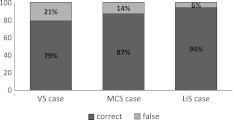
Diagnostic accuracy as studied by three case vignettes on the vegetative state (VS), minimally conscious state (MCS), and locked-in syndrome (LiS). The *χ* ^2^ test over all cases was significant (*p* < 0.001). *N* = 503, VS case (*N* = 168), MCS case (*N* = 171), LiS case (*N* = 164); numbers may not add up to 100 due to rounding

Overall, 86% (*n* = 434) of the participants chose the correct diagnostic category. Of those participants who evaluated the VS case, 79% gave the diagnosis VS, 18% chose the diagnosis MCS, one participant chose coma (1%), and 2% chose LiS. Of the participants who received the MCS case, 87% gave the diagnosis MCS, 4% chose VS, 7% chose LiS, and 2% chose the option to suggest an alternative diagnosis (e.g., severe anoxic brain injury). Participants with the LiS case chose the diagnosis LiS (94%), MCS (4%), VS (2%), and one participant chose the option “other.” The rate of accuracy differed significantly (*p* < 0.001) according to the three case vignettes (see Fig. 1).

In total, 83% of the female participants compared to 88% of the male participants accurately diagnosed the cases (*p* = 0.14). Participants who erred were slightly less certain about their diagnostic accuracy than participants who gave the correct answer (median 8 vs. 9 on a NRS, *p* < 0.001).

#### Professional experience in years, with patients with DOC or in rehabilitation care

Neurologists with more years of professional experience had a slightly lower chance of misdiagnosing the MCS case (OR for 1 year more: 0.9; CI 0.9–1.0; *p* < 0.05), but professional experience had no significant influence on the accuracy of the neurologists’ application of the diagnostic categories to the VS or LiS vignettes (*p* = 0.23, *p* = 0.69).

Of the participants who received the VS case and gave information on their experience with VS (*n* = 159, missing data: *n* = 9), 76% of the neurologists who cared for less than 20 VS patients, (*n* = 96; 60%) diagnosed the VS case accurately, not significantly different from the 84% of those who cared of more than 20 patients (*n* = 63, 40%; *p* = 0.22). However, in the group of participants who received the MCS case (*n* = 164, missing data: *n* = 7), 93% of those who were more experienced in the care of VS patients (*n* = 74, 45%) chose the correct diagnosis compared to 82% (*n* = 90, 55%) of those who were less experienced (*p* = 0.04). In the VS case, 79% of the participants who were more experienced in the care of MCS patients (*n* = 56, 36%) chose the accurate diagnosis, being equal to the group of participants who were less experienced in the care of MCS patients (*n* = 99, 64%; *p* = 0.975). Yet, being highly experienced in the care of MCS patients was helpful in choosing the right diagnostic category for the MCS patient (*n* = 159, missing data: 12). Out of those who had cared for more than 20 patients (*n* = 64, 40%) 95% diagnosed the case accurately compared to 84% of those who cared for less than 20 patients (*p* = 0.03). The accuracy rates of those working in a rehabilitation setting (*n* = 107) for the patients in VS (72%), MCS (89%), or LiS (91%) did not significantly differ from the accuracy rate of those who did not work in a rehabilitation setting (80%, 86%, 95%; *p* = 0.30; *p* = 0.64; *p* = 0.49).

We continued our data analysis with participants who accurately applied the diagnostic knowledge to the cases (from now on referred to as the VS group: *n* = 132, MCS group: *n* = 148, and LiS group: *n* = 154).

#### Patients’ capabilities

The percentage of neurologists agreeing with the presence of cognitive, emotional, and behavioral capabilities in the described patients are displayed in Table 3. Neurologists had different beliefs about which capabilities patients with VS, MCS, and LiS have. Disagreement was highest in the MCS case. Neurologists generally agreed that patients like the LiS patient were aware of themselves and their surroundings, and that the VS patients were not. Half of the neurologists agreed that MCS patients were aware; half of them did not. Within the respective cases, however, we found inconsistent answers. Most neurologists thought that VS patients are not aware, yet a large proportion of them simultaneously stated that VS patients feel pain and experience hunger and thirst. Only 61% of neurologists thought that LiS patients could feel touch.

**Table 3:** Frequency of agreement with capabilities of a patient in the respective condition as judged by neurologists

Frequency (%)	VS group (*n* = 132)^a^	MCS group (*n* = 148)^a^	LiS group (*n* = 154)^a^
Being aware of themselves	9	54	94
Being aware of surroundings	6	57	94
Feeling pain	77	96	86
Smelling odors	35	78	85
Tasting flavor of food/drinks	29	77	63
Feeling touch	67	94	61
Having emotions	35	87	93
Recognizing their name	12	67	92
Recognizing people	13	85	95
Experiencing hunger/thirst	46	92	83
Having sexual desires	13	47	68
Understanding what others say	8	39	93
Having thoughts	23	72	97
Experiencing dreams	36	76	90
Remembering experiences	13	54	92
Storing new information	8	32	85
Expressing desires	2	20	70
Interacting with others	8	57	86

*VS* vegetative state, *MCS* minimally conscious state, *LiS* locked-in syndrome

^a^Those who correctly diagnosed the patients in the respective cases

### Attitudes toward limitation of life-sustaining treatment

**Fig. 2:**
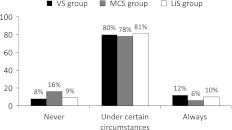
Attitudes of those participants who accurately diagnosed the respective cases toward the limitation of life-sustaining treatment: “In the prior case life-sustaining treatment should be limited…?” Overall there was a trend toward significant differences (*p* = 0.09). Differences between the attitudes for VS and MCS are statistically significant (*p* = 0.04, χ^2^ test). *N* = 434, VS group (*N* =132), MCS group (*N* = 148), LiS group (*N* = 154); missing data: VS group *n* = 1; MCS group *n* = 1, LiS group *n* = 5

The frequencies of whether LST should be limited for the patient in the case vignette are presented in Fig. 2. The attitudes did not differ among the three cases of VS, MCS, and LiS, but there was a statistical trend (*p* = 0.09). While there were no significant differences between the VS and the LiS group (*p* = 0.82) or the MCS and the LiS group (*p* = 0.11), fewer participants would limit LST in the MCS case compared to the VS case (*p* = 0.04).

#### Circumstances in support of limiting LST

Table 4 shows how many participants agreed with limiting LST under certain circumstances, with the agreement scaled on a five-point rating scale. The particular circumstances led to differing attitudes toward limiting LST. Note that the participants who said they would never limit LST according to the case in the prior question skipped this question and the option “does not apply” was provided for the circumstances that did not apply to the patient in the respective case (such as no chance of recovery of consciousness to the LiS case).

**Table 4:** Distribution and level of agreement (in %) with limiting life-sustaining treatment under certain circumstances depending on the case groups

Agreement (%)				Rating					
				1 = extremely weak, 5 = extremely strong					
Circumstances	Groups^a^	Median	*n*	1	2	3	4	5	*p*
Patient’s will is opposed to LST	VS	5	116	1	1	3	11	85	0.04
	MCS	5	121	1	3	4	22	70	
	LiS	5	134	2	1	3	19	75	
Patient suffers additional fatal disease (e.g., cancer)	VS	5	116	3	4	5	20	68	0.05
	MCS	5	118	0	6	8	29	57	
	LiS	5	128	4	7	12	23	55	
Surrogate decision maker refuses consent to LST	VS	4	117	1	11	22	31	35	0.001
	MCS	4	119	9	12	20	36	24	
	LiS	4	132	10	13	27	38	13	
No improvement after 1 year or longer	VS	4	107	10	11	25	23	30	<0.001
	MCS	3	115	20	18	22	25	15	
	LiS	2	123	26	29	24	15	7	
No chance for recovery of consciousness^b^	VS	4	111	5	14	15	23	43	0.06
	MCS	4	115	8	15	16	26	36	
	LiS	4	123	19	8	16	29	28	
No chance for recovery of communication²	VS	3	108	7	19	27	27	20	0.01
	MCS	3	116	13	23	30	20	15	
	LiS	3	107	22	16	32	18	12	
Patient obviously suffers intensely	VS	3	109	8	15	31	28	17	0.19
	MCS	3	117	11	20	26	31	13	
	LiS	2	127	13	22	25	28	12	
If elderly (e.g., 70 years or older)	VS	3	102	18	28	30	17	7	0.003
	MCS	3	115	16	24	26	23	12	
	LiS	2	116	29	28	25	12	6	
No chance for recovery without disability	VS	2	103	44	27	15	9	6	0.55
	MCS	2	111	47	25	23	3	2	
	LiS	1	121	51	24	11	11	3	
Resources are scarce and costs high	VS	2	100	46	30	17	5	2	0.03
	MCS	1	113	55	21	14	6	4	
	LiS	1	116	65	21	10	3	2	

*LST* Life-sustaining-treatment

From left to right: circumstances under which ^a^ those who correctly diagnosed the patients in the respective cases (VS group: *n* = 132, MCS group: *n* = 148, and LiS group: *n* = 154) agree with limiting LST; *N* numbers of participants who rated the agreement with LST under specific circumstances, frequency of participants (in %) who chose the respective number

^b^If circumstances do not apply to the case (here to LiS), participants could choose “does not apply.” Kruskal-Wallis test; numbers may not add to 100 due to rounding

Overall, fewer female participants who correctly diagnosed the cases (*n* = 115) gave the answer to never (9%) or always limit (4%) LST than did male participants (*n* = 292; 12%, 12%; *p* < 0.05). There were significant gender differences in the willingness to limit LST under certain circumstances (see Table 5). More men than women agreed extremely strongly to limit LST if the patient suffers from an additional fatal disease or if there is no chance to recover communication. Yet more women agreed to limit LST if resources were scarce and costs were high.

**Table 5:** Distribution and level of agreement (in %) with limiting life-sustaining treatment under certain circumstances depending on the participant’s gender

Agreement to limit LST:				1 = extremely weak, 5 = extremely strong					
Circumstances	Gender^a^	Median	*N*	1 (%)	2 (%)	3 (%)	4 (%)	5 (%)	*p*
Patient’s will is opposed to LST	Male	5	251	2	1	2	16	79	0.08
	Female	5	104	1	2	6	21	70	
Patient suffers from additional fatal disease	Male	5	252	2	4	8	24	62	0.03
	Female	5	100	3	10	10	26	51	
Surrogate decision maker refuses consent to LST	Male	4	249	6	13	23	35	24	0.76
	Female	4	98	8	9	27	34	22	
No improvement after 1 year or longer	Male	3	237	19	20	22	21	19	0.46
	Female	3	93	20	18	27	22	13	
No chance for recovery of consciousness^b^	Male	4	221	9	12	16	24	39	0.19
	Female	4	92	13	13	14	30	30	
No chance for recovery of communication^b^	Male	3	225	12	15	30	26	17	<0.001
	Female	3	93	22	28	29	10	12	
Patient obviously suffers intensely	Male	3	249	12	19	28	29	12	0.33
	Female	3	98	10	19	24	30	17	
If elderly (e.g., 70 years or older)	Male	3	231	23	25	28	17	8	0.62
	Female	3	89	18	30	25	19	8	
No chance for recovery without disability	Male	2	234	45	26	16	9	5	0.21
	Female	1,5	88	50	26	18	5	1	
Resources are scarce and costs high	Male	1	228	60	21	14	4	2	0.002
	Female	2	88	40	33	15	8	5	

*LST* life-sustaining treatment

From left to right: circumstances under which ^a^ those who correctly diagnosed the patients in the respective cases and are male (*N* = 292) or female (*N* = 116) agreed with limiting LST; *N* numbers of participants who rated their agreement with LST under specific circumstances. Values in the table represent the distribution of participant’s responses on the rating scale

^b^If circumstances do not apply to the case (here to LiS), participants could choose “does not apply.” Mann-Whitney *U* test; numbers may not add to 100 due to rounding

#### Treatment measures

**Fig. 3:**
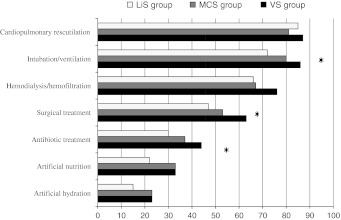
Forms of life-sustaining treatment that neurologists who gave the accurate diagnosis would consider limiting (under certain circumstances or always). The *bars* indicate the percentage of respondents in each diagnostic group: VS (*black*), MCS (*grey*), and LiS (*white*). *n* = 434, VS group (*n* = 132), MCS group (*n* = 148), LiS group (*n* = 154);* asterisks* significant differences among the three groups of respondents to the cases, using Pearson’s χ^2^ test (*p* < 0.05; intubation/ventilation: *p* = 0.02, surgical treatment: *p* = 0.02, antibiotic treatment: *p* < 0.05)

Figure 3 shows the frequency of agreement with the limitation of particular treatment measures. For most measures, the readiness to limit treatment was highest in the VS group, lower in the MCS group, and lowest in the LiS group. Significant differences between the three groups concerned artificial respiration (*p* = 0.02), surgery (*p* = 0.02), and administration of antibiotics (*p* < 0.05).

Participants who correctly diagnosed the cases were also asked whether they would make the same decisions for themselves if they were in the situation of the patient in the case vignette (*n* = 417). The majority (71%) would want the same decisions to be made for them, 29% would favor less intensive LST measures, and only 1% would choose more intensive LST for them than they considered for the patient in the case vignette.

Significantly fewer female neurologists were willing to limit the use of antibiotics (30%), artificial nutrition (22%), and artificial hydration (10%) than were male neurologists (40%; 33%; 25%; *p* = 0.047, *p* = 0.032, *p* = 0.01). There were no significant gender differences in regard to the other therapeutic measures (cardiopulmonary resuscitation: 86 vs. 88%, *p* = 0.60; intubation and ventilation: 81 vs. 78%, *p* = 0.53; hemodialysis: 71 vs. 96%, *p* = 0.70; surgery: 57 vs. 52%, *p* = 0.38).

#### Quality of life, prognosis, religion, and attitudes toward LST

The option “I don’t feel able to rate the patient’s quality of life” was chosen by 32% of the participants (*n* = 172), the lowest amount being in the VS group (28%), followed by the LiS (38%), and MCS (39%) group. The option that the patient had no quality of life was chosen by 14% in the VS group, 1% in the MCS group, and 3% in the LiS group. The median quality of life of the VS patient (1; span 0–4) and LiS patient (1; span 0–9) was rated lower than that of the MCS patient (2; span 0–7), and the three groups differed significantly (*p* < 0.001).

Only 3% of those who accurately diagnosed the VS case and 10% of those who diagnosed the LiS expected a better outcome (modified Rankin scale 4-0) compared to 25% of those who correctly diagnosed the MCS case (*p* < 0.001). The others expected severe disability (modified Rankin scale 5).

Of those who practiced a religion and diagnosed the patient correctly (n=213), 6% would always limit LST in the respective case, 13% never, and 81% would limit treatment under circumstances, significantly different from those who did not practice a religion (*n* = 200), where 13% would always limit LST, 9% never, and 78% under certain circumstances (*p* = 0.03). Of those who had spiritual beliefs in the existence of god, and diagnosed the patient correctly (*n* = 274), 6% would always limit LST, 11% never, and 83% under circumstances. Of those who did not have spiritual beliefs, and diagnosed the case correctly (*n* = 136), 16% would always limit LST, 10% never, and 74% under certain circumstances (*p* = 0.01).

### Appraisal of ethical challenges

Table 6 displays how the participants judged different ethical challenges in the care of DOC patients. Prognosticating recovery and determining the patient’s wishes were seen as extremely challenging for all three conditions. The least challenging issues were reaching an agreement as a team and multidisciplinary discussions. Overall, there were only slight differences among the three conditions. Some of the aspects seem to be a bit more challenging in one condition than another one, such as applying a surrogate’s decision in VS compared to LiS cases.

**Table 6:** Appraisal of ethical challenges in the decision-making process for patients like the patient in the presented case

Median (1st, 3rd quartile) on NRS (0–10)	Missing data (*n*)^b^	VS group^a^	MCS group^a^	LiS group^a^	*p*
Making prognosis and predicting recovery	17	8 (7, 10)	9 (8, 10)	8 (6.25, 10)	0.12
Determining patient’s wishes	17	8 (7, 10)	8 (7, 10)	9 (8, 10)	0.03
Deciding for patient in absence of surrogate	18	8 (7, 10)	8 (6, 10)	8 (7, 10)	0.29
Discontinuing LST	19	8 (5, 10)	8 (5, 10)	7 (6, 10)	0.16
Making correct diagnosis	15	7 (4, 9)	7 (3.5, 8)	7 (3, 9)	0.58
Accompanying family members in decisions	17	7 (6, 9)	7 (5, 8)	8 (6, 9)	0.01
Applying a decision made by surrogate	20	7 (5, 8)	5 (4, 8)	7 (3, 9)	0.001
Evaluating resource allocation	26	7 (4, 9)	7 (5, 9)	6 (3, 9)	0.049
Assessing medical futility	21	7 (5, 8)	7 (5, 8)	7 (6, 8)	0.75
Finding long-term care	21	6 (4, 8)	6 (3, 8)	6 (3, 8)	0.46
Accompanying clients through staff rotations	21	6 (3.5, 8)	6 (5, 7)	6 (5, 9)	0.28
Multidisciplinary discussions for decisions	17	5 (3, 7)	5 (2, 8)	5 (3, 8)	0.39
Reaching an agreement as a team	17	5 (3, 7)	5 (3, 8)	6 (4, 8)	0.06

^a^Those who correctly diagnosed the patients in the respective cases; *n* = 434, VS group (*n* = 132), MCS group (*n* = 148), LiS group (*n* = 154); Kruskal-Wallis test

^b^Missing data: sum of all cases

## Discussion

In this study, we used case vignettes instead of commonly used diagnostic terms [13–15, 20, 21] to investigate neurologists’ attitudes toward ethical issues that arise in the care of DOC and LiS patients. The overall accuracy in the application of diagnostic knowledge of DOC patients was found to be high in our vignette-based survey (79–86.5%). Our approach can only be cautiously compared to studies verifying patients’ diagnoses by clinical assessment or structured neurobehavioral examination. To compare the accuracy rates, we should not refer to misdiagnosis of case vignettes, but to misdiagnosis of diagnostic categories: how many of those who diagnosed VS actually had the VS case? If we recalculate the data accordingly, misdiagnosis in our study was between 7% (VS) and 20% (MCS), which is lower than in the clinical studies (37–43% of the patients diagnosed as VS are actually in a MCS or other condition) [5, 6, 22]. This may be explained by the differing methodological approach of our study. We targeted neurologists instead of following up on patients and comparing diagnosis made on the basis of clinical assessment with diagnosis on the basis structural neurobehavioral tests. Furthermore, we assessed the application of diagnostic knowledge by presenting a typical, briefly described case and offering a limited range of potential diagnoses to choose from. In our study, the VS case led to the highest error rate, and the error that most often occurred was overestimating the patient as being in a MCS, suggesting the need for greater training in distinguishing patients on the borderline of VS and MCS. The error of overestimating patients in the VS as being in the MCS was not described by Schnakers et al. [22]. One could argue that overestimating the patient’s capabilities is an error that is more preferable than underestimating them when considering the best interest of the patient. However, the consequences could be positive and negative: more rehabilitation treatment could be provided to the patient and a longer timeframe could be awaited to declare the condition as chronic. If the patient had anticipated a decision for the VS his autonomy would not be respected because of the misdiagnosis. Furthermore, the patients’ family could develop unrealistic expectations for recovery, communication, or other outcomes, and it would cause expenses for ineffective treatment. We were unable to determine factors that reduce the probability for misdiagnosis for the VS case, but for the MCS case experience with patients and years of professional experience were of value, which suggests including exposure to these sorts of patients during training. That experience did not predict a more accurate application of diagnostic knowledge for the VS case, but for the MCS case, is surprising, and perhaps the VS case stating that the patient “inconsistently” follows commands was misleading. Although neurologists who gave the correct diagnosis were significantly more confident and certain about their diagnosis than those who erred, even the latter had a relatively high level of confidence in their diagnostic skills. This may be problematic because they will probably not seek further training or a second opinion.

The assessment of the patients’ remaining capabilities (Table 3) provided insight into the neurologists’ understanding of VS, MCS, and LiS. This transcends the task of applying a diagnostic category to a case vignette and touches on beliefs of what it is like to be in such a state [23]. According to the traditional medical concept, VS patients are not aware, MCS patients have a rudimentary, partial, or inconstant awareness, and LiS patients are fully aware of themselves and their surroundings [24]. The responses reflect this distinction, with only a small percentage of neurologists who believe that VS patients are aware and a small percentage that even negates awareness in the LiS. The neurologists in the MCS group are divided: half of them regard the described MCS patient as being aware and the other half does not. This may reflect the breadth of and the uncertainty about the clinical continuum that covers the category of MCS, which has recently been subdivided into MCS plus (with high-level behavioral responses like command following) and MCS minus (with low-level behavioral responses like visual pursuit) [25]. The results suggest that the evaluation of the patient’s remaining capabilities is highly dependent on the neurologists’ conceptual understanding of the syndrome. Interestingly, although awareness is usually regarded as a prerequisite to all other mental phenomena, many participants agreed that VS patients were unaware, but still thought that they were able to dream, have thoughts and emotions, and perceive gustatory and tactile stimuli, including pain. Studies using somatosensory-evoked potentials and positron emission tomography have shown that noxious stimuli activate the pain matrix in MCS patients just like in controls, but in VS patients it is activated to a far lesser extent and without functional connectivity [26, 27]. Consistent with these findings, almost all neurologists in our survey agreed that MCS patients experience pain. Yet, more than three quarters of participants assume that VS patients also experience pain and nearly half of them assume that VS patients also feel hunger and thirst. In the survey by Demertzi et al. [28] a lower number (56% of the medical doctors) assumed that VS patients feel pain while an equally high number affirmed pain perception for MCS (96%). Experience with patients’ motor or vegetative reactions to noxious stimuli might have influenced the neurologists’ assumptions. Such reactions are not necessarily a sign of conscious awareness, which is a prerequisite for the experience of pain. In a survey among LiS patients who were in a chronic condition (at least 1 year after a brainstem vascular accident), half of the participants experienced pain and two-thirds anxiety [29]. More studies in this area are clearly warranted. It would be interesting to compare neurologists’ beliefs about the capabilities of LiS patients with the patients’ self-assessments.

Over 90% of the participants would consider limiting LST for the VS and LiS patient at least under certain circumstances, but only 84% would consider it for MCS patients. Contrary to our expectations, there were no statistically significant differences in the attitudes toward limitation of LST among all three cases. The difference between MCS and VS is in agreement with the recent European survey done by Demertzi et al. [17], yet they found far lower rates of physicians’ (i.e., medical professionals) agreement with treatment limitation (67% for VS and 27% for MCS). The difference between the attitudes toward VS and MCS when selecting the attitudes of the participants from central Europe was still much larger than in our survey. While their survey asked about patients more than 1 year after injury, our vignettes referred to patients with non-traumatic injuries that occurred only 4 months prior. The use of case vignettes instead of diagnostic terms and the sample of clinical neurologists instead of attendees of scientific conferences might explain the different results of the two studies. Comparing our results to other surveys, we have to differentiate between attitudes toward treatment limitation in general or toward withdrawal of artificial nutrition and hydration in particular. Our results on VS are quite similar to the results of American, British, and Belgian surveys from the 1990s when comparing the willingness to limit treatment in general (88–91% agreement to limit LST), but our participants were less willing to withdraw artificial nutrition and hydration [13–15]. Only 34% of our participants, who accurately diagnosed the VS case, would withdraw artificial nutrition and only 23% would withdraw artificial hydration in the VS case. In the American, British, and Belgian surveys, 56–89% considered the withdrawal of artificial nutrition and hydration appropriate. Even in an Italian survey from 2011 a higher percentage of the physicians (66%) believed that the withdrawal of artificial nutrition and hydration to be appropriate depending on the patient’s wish [30]. On the other hand, surveys from Japan, and an older German survey of 283 medical directors of neurological, neurosurgical, and rehabilitation departments reported much lower rates of agreement with limitation of treatment (30, 58%) and withdrawal of artificial nutrition and hydration (3, 16%) [16, 20, 31]. Lanzerath et al. gave two reasons for this: (1) in Germany, artificial nutrition and hydration were considered to be a form of indispensable basic care and not a medical treatment measure that could be withdrawn; (2) the experience with the misuse of medicine during National Socialism has led to a higher sensibility for ethical concerns, especially regarding end-of-life decisions. There might also be additional reasons. If the law in a country explicitly allows limiting artificial nutrition and hydration in VS patients, like in Great Britain after the case of Tony Bland, this might influence the physicians’ attitudes. Moreover, religiosity is a factor that influences treatment decisions [17], which was confirmed by our results. Another explanation could be found at the level of methods: most questionnaires provided only two options (yes or no), while we offered three answer alternatives and most participants chose the option “under certain circumstances.” Although the sample of the German survey from 1997 is not directly equivalent to our sample, it can be hypothesized that the attitudes of neurologists have become more liberal since then, paralleling a process of liberalization in German medical law and ethics [32, 33].

The similar results on the attitudes toward LST in the VS and LiS group warrant the conclusion that the presence or absence of consciousness does not seem to be the basis for neurologists’ decisions to limit LST, which is consistent with the arguments of Levy and Savulescu and Wilkinson et al. [34, 35]. It is surprising that so many neurologists agree with limiting LST for patients in the LiS. Lulé et al. [36] reported that despite their extreme motor impairment, a significant number of LiS patients maintain a good quality of life that seems unrelated to their state of physical functioning. In the study by Bruno et al. [29] 58% of the LiS patients declared they did not wish to be resuscitated in case of cardiac arrest, and 53% had envisaged euthanasia, but only 7% had a current wish for euthanasia. The authors identified satisfied and dissatisfied subpopulations that also differed in symptoms of depression, anxiety, and in the time they had spent in the LiS. A delay of end-of-life decisions was suggested to allow patients with more recent injuries to adapt psychologically to the new situation. In our survey, the median estimated quality of life of the LiS patient was as high as the median quality of life of the VS patient. Prognosis of functional outcome could explain the decision for or against treatment limitation for the MCS case. Patients in the MCS are regarded as having a better prognosis than VS patients [37], but studies that examine the prognosis of MCS patients prospectively are rare [38], and clinicians often refer to single cases of remarkable late recoveries of MCS patients [11]. We showed that female participants tend to be more uniform in their attitudes (preferring treatment limitation under certain circumstances instead of “always” or “never”). In other surveys male gender was significantly associated with greater willingness to forego LST [39] and to discuss end-of-life decisions with competent patients [40]. The authors of the first study argued that female physicians were following care ethics rather than rights-based ethics.

There were more differences among the three cases when it came to the agreement with limiting LST under given circumstances and the concrete treatment measures. One of the strongest circumstances that led to agreement for limiting LST was that the patient’s will opposed treatment. This is in accordance with the ethical principle of respect of patient autonomy [41]. Interestingly, patient autonomy was more often extremely strongly agreed upon in the VS case than in the MCS or LiS case, suggesting that indirectly diagnosis plays a role in the actualization of the patient’s will. In German law, a patient’s advance directive has to be respected. The refusal of treatment by the patient’s surrogate decision maker is also binding [33]. The surrogate, however, has the obligation to decide according to the patient’s will, yet in practice the patient’s will might be overruled by other arguments such as the surrogate’s expectation of the patient’s recovery [42]. In our study, there was strong agreement with the limitation of treatment if the surrogate refuses consent, particularly in the VS case. No improvement for more than 1 year was a stronger argument in the care of a VS patient, a mediocre argument in the care of a MCS patient, and a weaker argument in the care of a LiS patient. Opinions on the VS were consistent with conclusions of expert groups who consider VS as permanent 3 or 6 months after non-traumatic brain injury or a year after traumatic brain injury [43, 44]. Yet, the recommendations were criticized for including patients in their survival data who died primarily because their life-sustaining therapy was discontinued [38]. When the recommendations about the VS were made, the MCS had not been recognized as a diagnostic category, yet and no time frames are known to declare the MCS to be irreversible. Women were not in general less against treatment withdrawal than men. They agreed less than men with limiting treatment for patients who suffered from an additional fatal disease or who had no chance for recovery of communication, but they agreed more with limiting treatment if resources were scarce and costs high.

Age and resource scarcity were considered to influence LST decisions only slightly. We know from specific surveys and qualitative studies that these factors do indeed influence treatment decision making [45, 46]. Such factors may act implicitly (as a cause) but may not be explicitly acknowledged (as a reason). There is a tendency to answer these questions according to social desirability, and both ageism and rationing due to resource scarcity are still taboos in Germany.

Most participants considered forgoing cardiopulmonary resuscitation and mechanical ventilation. They were, however, reluctant to limit artificial nutrition or hydration. This pattern is well known from many studies on attitudes and actual practices of end-of-life decision-making [47, 48], including a recent survey of German intensive care clinicians [49]. We identified gender differences in the willingness to withhold antibiotic treatment, artificial nutrition and hydration. More women were reluctant to limit these treatment forms than men. To our knowledge, gender difference particularly in regard to the willingness to withdraw artificial and hydration in disorders of consciousness patients have not been described before. Decisions about artificial nutrition and hydration in DOC patients are still controversial issues for healthcare providers both in Europe and North America [10, 12]. Most countries’ laws, including German law, do not differentiate between withdrawing or withholding artificial nutrition and hydration or any other form of LST.

That the majority of the neurologists would prefer the same treatment measures for themselves can be interpreted in two ways. It could be claimed that their decisions are value-driven or that they want the best treatment for their patients as they would want for themselves. Interestingly almost no one would prefer more treatment for themselves than for the patient, but 30% preferred more treatment for the patient than for themselves. Maybe they want to be more cautious about their patients than about themselves or perhaps to avoid feelings of guilt.

We found that determining the prognosis of potential recovery as well as determining the patient’s wishes regarding the treatment in conditions such as VS, MCS, and LiS were the most ethically challenging issues for the participants of this study. This finding was not surprising given that the challenge of prognosis is a recurring theme in the literature on DOC, as well as in the care of severely ill neurological patients on the whole [10]. Identifying patients’ treatment wishes is known to be one of the most difficult challenges for physicians, especially if the patients are incompetent and uncommunicative [50]. Advance directives may facilitate decisions in accordance with the patients’ wishes, but as brain injuries are mostly unexpected and often strike young, healthy people, advance directives are rarely present for DOC patients. Even if they are available, their interpretation can be difficult [51]. Issues that were judged to be less challenging (e.g., finding long-term care placements, accompany families and treating patients within staff rotations, discussing treatment decisions in a multidisciplinary context) revolve around contextual or institutional factors. Contextual factors have been shown in different studies to influence physicians’ decisions [52], but physicians may not be cognizant of them or fully acknowledge them [53]. Our results reflect how physicians themselves understand ethical challenges, which may not be congruent with their own practices or with the attitudes of family members or other healthcare providers [54]. The online survey provides an efficient and successful method for the measurement of knowledge and attitudes toward decision making for DOC patients that can be conducted in different countries, with physicians with different subspecialties and with members of other professions (e.g., nurses).

## Limitations

Our e-mail survey had a moderate response rate, low in comparison with mailed surveys [55]. The fact that online surveys have lower response rates than mailed surveys is in accordance with a previous study with residents and faculty [56]. Compared to other online surveys with physicians, our response rate is acceptable (other surveys: 5% [57], 13%, [58], 72% of 68 active members of a professional society [59]). Our survey was relatively long, which may have put off potential participants. The society we accessed has over 6,000 members, and therefore diffusion of responsibility might also lower the response rate. Another problem was that we recruited during summer holidays. Some potential participants informed us that they did not fill in the questionnaire because they did not care for DOC patients. In spite of these caveats, the cohort in our study was still representative for the German Society of Neurology by age and region, and the sample representative by age, but not by gender. Statistical analysis showed that gender had no influence on diagnostic accuracy, but it had an influence on the attitudes toward the limitation of LST. It is possible that our survey overrepresented the perspective of male neurologists that seem to be more in favor of extreme answers (to always or never limit LST), as compared to the female participants considering treatment limitation under certain circumstances. To fully acknowledge gender differences in the attitudes toward treatment limitation the issue should be in the focus of following studies.

Members of other specialties such as anesthesiology, palliative medicine, rehabilitation medicine, and pediatrics were not included given our focus on neurology experts and our goal of measuring the application of diagnostic knowledge. These specialists could be included in further studies to understand differences between specialty physicians as well as differences between physicians and other healthcare providers. Other studies revealed that there are cultural differences in the attitudes toward treatment limitation; therefore, our results should be compared to those of other countries [60].

## Conclusion

The application of diagnostic knowledge of VS, MCS, and LiS was accurately performed by most German neurologists. Their attitudes were mostly in favor of limiting life-sustaining treatment and slightly differed between MCS as compared to VS and LiS. Attitudes strongly differed under certain circumstances (e.g., patient’s will opposed treatment) and according to treatment measures.
